# Social license -What’s in a name?

**DOI:** 10.3389/fbioe.2024.1395445

**Published:** 2024-06-25

**Authors:** Jeantine E. Lunshof

**Affiliations:** Wyss Institute for Biologically Inspired Engineering, Harvard University, Boston, MA, United States

**Keywords:** bioinnovation, sustainability, social license, mining, stakeholders, ethics, responsible innovation, Ubuntu

## Introduction

### Should social license be used in sustainable bioinnovation?

Putting the question in context, all three notions, Social License, sustainability, and bioinnovation are trending and all three sound good, suggesting actions and goals worth pursuing (Purvis et al., 2019). However, that does not mean that Social License is necessary, or even desirable, for successful and responsible sustainable bioinnovation. Taking a closer look at the terminology: sustainability is a concept with a long history, dating back to at least the sixties and seventies of the 20th century, and it is used with different meanings in diverse contexts (Purvis et al., 2019). Biotechnology innovation has been advocated for in a concerted effort since 1993 (BIO Biotechnology Innovation Organization, 1993), and it involves all fields of biotechnology and related areas in, for example, medicine, economics, law, governance, and regulatory sciences. In this short essay I will zoom in on the notion and practice of Social License and discuss whether it is an appropriate instrument to achieve the goals of sustainable bioinnovation, using an example from synthetic biology.

## Social license, what’s in a name?

“Social License” is short for “Social License to Operate” (SLO) and this notion originally arose in the context of extractive industries—mining—and later became wider used for interventions in natural ecosystems or in social systems, as, for example, public health practices, or even in a combination of those spheres: the assumed license to carry out programs for wide-area vector control. I will get back to that use case further below.

In 1997, James Cooney, executive director of a gold mining company, discussed the problem of “obtaining social license to operate” in a discussion with the World bank (Gehman et al., 2017). Two industry consultants, Joyce and Thomson, developed the concept of SLO further, and added the aspects of legitimacy, credibility, and trust. Their foundational definition reads:

“A social license to operate exists when a mineral exploration or mining project is seen as having the approval, the broad acceptance of society to conduct its activities. Such acceptability must be achieved on many levels, but it must begin with, and be firmly grounded in, the social acceptance of the resource development by local communities” (Joyce and Thomson, 2000).

This definition sounds good, in the moral sense, emphasizing “approval,” “acceptability, “social acceptance,” but this may be too good to be true if we take the context into account: extractive industries. In other words, an outside third party strives to be granted permission for operations that are their primary for-profit business interest. In this setting, neither the “social” nor the “license” refer to moral values or altruism. Is Social License to Operate “a term largely invented by business for business,” as Gehman et al., 2017 suggest? Similarly, Kemp and Owen, 2013, in their thorough critical analysis, conclude “Even through an appreciative read, social license remains a pragmatic calculation of what is required to minimize business risk and win the degree of community support required to avoid delay or disruption to company operations”.

Social License was further developed and put into practice in mining projects by Thomson and Boutilier, and they founded socialicense.com (The Social License to Operate, 2020). The widely used “Social License Pyramid” visualizes the “conversion” of a community from initial refusal to allow mining activities on or under their land, to not only approval, but above and beyond that psychological identification with the third party exploitation of their resources. This strategy raises serious ethical, social, legal, and political concerns and has been addressed in detail by Kemp and Owen, 2013, Gehman et al., 2017, and many others.

While the criticism above refers to the use of the term Social License to Operate in mining, and to similarly extractive activities like logging, we need to ask whether the terminology should be adopted in responsible bioinnovation, including projects that aim at providing populations with improved crops or pursue public health goals, as is the case in area-wide vector control, for example, control of malaria or dengue transmission through genetic interventions in mosquitos.

## Social license in bioinnovation

In a recent study on the use of “Social License” in synthetic biology (Delborne et al., 2020), we conducted a systematic review of the literature until mid 2019, and found increasing use of “social license to operate” in peer reviewed and non-peer reviewed literature in the synbio field from 2015 till 2019. Similar to the use in the mining context, obtaining social license was used to describe the societal buy-in necessary for the development and use of novel synthetic biology-based technologies. A first reference we found, was in a 2015 meeting report of the de-extinction-focused organization Revive&Restore (Revive and Restore, 2015). We encountered the introduction of this term known from the extractive industries while being involved in research on the social and ethical aspects of gene drives, a novel and highly controversial bioinnovation. A gene drive is a construct occurring naturally or achieved through genetic engineering that enables a trait to spread through a population of animals or plants with higher than Mendelian probability (James et al., 2023). That means that traits of a species—for example, transmitting malaria—can be overpowered or even erased. Not all gene drive research and development is for-profit, and many projects are enabled by philanthropic funding, for example, Target Malaria (Target Malaria, 2021).

**FIGURE 1:**
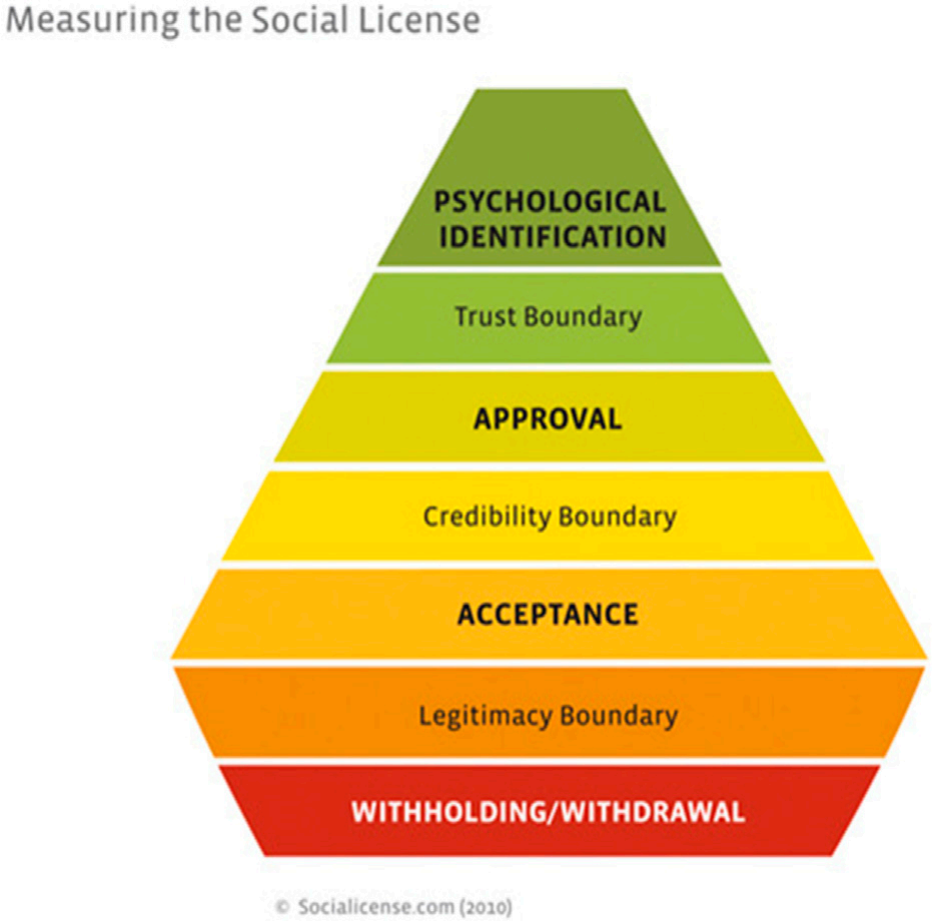
Measuring the social license.

One specific feature of gene drives, and of many applications of genetically modified organisms, is that their intended use is in the shared environment and self-propagation in an ecosystem, irrespective of local sovereignty or national borders. And exactly that raises the question of acceptability, and of community consent: who benefits and who is affected by potential harms? Like the case of mining, the question is whose interests are served and who decides about initiating the activity—here the deployment of the genetic constructs.

It is tempting to introduce the concept of “Social License”: if successfully obtained, it would change attitudes of the involved population from refusal to embracing the technology and even psychological identification. At least, that is the idea.

The reality, however, is far more complex and there are important differences between mining operations and bioinnovation projects. Deciding about the introduction of synthetic biology-based ecosystem interventions is complex due to a number of features that are inherent to these technologies. These features are different from those in mining. In mining, estimates about the effects on the environment are possible, based on very longtime experience: evidence of earliest mining activities in Ngwenya dates from 43.000 years BC (UNESCO World Heritage Convention, 2008). The many forms of lasting damage to the environment from modern mining are well-documented, mitigation and environmental remediation are lagging behind (MIT, 2016).

Synthetic biology-based ecosystem interventions are new and while *in silico* simulations yield probabilities about the effects—both the intended beneficial outcomes and the potential adverse effects—these remain probabilities. Inherent system effects make predictions difficult: all ecosystems are part of other, larger ecosystems and the scale of extent over time is hard to predict. While reversibility is often intended and is, for example, one of the criteria for gene drive release, it is unknown whether this will be the case indeed. After self-propagating organisms have been released in the wild the further course of events may be hard to influence. The fact that these organisms will cross geographic and national boundaries adds a complication, also in terms of international law. At the same time, the goal of the interventions is to realize the benefits of sustainable bioinnovation to communities and populations.

How can these technologies be introduced in a responsible manner, doing justice to humans and the environment? In my opinion, it cannot be Social License. A model that is inclusive, proactive, integrative, (it still has to be developed and proven) would rather focus on “Social Agreement” and not Social License. There is a substantial difference between reaching agreement or giving/obtaining a license.

## Bioinnovation—a window of opportunity

While novel bioinnovation projects are still at early stages of implementation—or even still at the stage of a research setting, as, for example, large cage experiments in gene drive mosquito research—there is a unique window of opportunity to consider possible scenarios for responsible deployment that include all stakeholders.

First, the stakeholders: who are stakeholders in the development of novel technologies that will be deployed in the shared environment?

The potential impact of synthetic biology-based ecosystem interventions could ultimately be global, but does that mean that the global community—if we can even say who they are—have a stake and should have a voice in decision-making? It is an easy claim that sounds good, but it does not recognize that stakes are unequal, some people have larger interests at stake than others. With these technologies it is fair to say that the communities where the technologies are actually being introduced and used have the weightiest interests. They may also receive the largest benefits, like a gene drive-based technology to curb the spread of malaria benefits the people in the regions where malaria occurs. In order to reap those benefits, these communities also bear the risk of possible adverse effects, if any would occur. As with biomedical and public health innovation, to be ethical and qualify as responsible innovation, four criteria must be met: a technology must be Available, Accessible, Acceptable, and Affordable for the users and other stakeholders (the 4A Framework^1^).

The 4A Framework is also a benchmarking tool during research and development. Genuine involvement of the primary stakeholders can already start at the stage of research, setting the research goals in alignment with the needs of the communities who will be the users. The likelihood that a technology is acceptable—socially, culturally—is far greater when the future users are involved at the stage of development. Accessibility, for example, deployment logistics, and affordability will usually depend on higher level structures in a society and those may be a bigger bottleneck in the innovation process. The 4A Framework thus outlines a mixed bottom-up and top-down model.

Communities can proactively seek solutions for their problem, thereby driving research. This is a key difference with the Social License model that is driven by third party—economic—interests to which communities should ultimately agree through a reward-oriented strategy. Social License, notwithstanding the pyramid visualization, is a top-down model. Moreover, history (extensively described by Boutilier and Thomson (2019) in the story of the San Cristobal mine) has shown that the “original” Social License is very difficult to obtain and even more difficult to sustain. Therefore, it may not even be worth pursuing this concept in sustainable bioinnovation. The big question is what the alternative can be. Understanding why not Social License is a first step. The key question in bioinnovation, also from an ethics point of view, is where does innovation or the wish for innovation start, who is the initiator and what is the motivation. The next question is about decision making, who decides and how are decisions made in, e.g., “co-production.” Co-production is aspirational, what is the next step to make it a reality? There is no clear answer/solution that is universally valid.

Examples of approaches to bioinnovation with early community and other stakeholder involvement have been the Genetic Biocontrol of Invasive Rodents (GBIRd) project, a global partnership of seven organizations including researchers at North Carolina State University, ATM University, the University of Adelaide, CSIRO (Australia), and the USDA (GBIRd Genetic, 2017).

The Responsive Science initiative, led by a team at MIT Media Lab (of which the author was a member), featured proactive open interaction between researchers and communities, interaction from the earliest stages of ideas and project design, and “adaptive science”—ongoing improvements in research based on new scientific insights and community input. Pilot studies, however, showed some of the real-world obstacles (Najjar et al., 2017; Buchthal et al., 2019; Normandin et al., 2022).

A different approach is found in communitarian values-based models, in the African context, for example, the use of Ubuntu, setting goals, methods and modes of interaction in alignment with regional values. A central element of Ubuntu is the interdependence of the interests of individuals and communities—in their environment—and their reciprocal obligations (Sambala et al., 2020; Shozi and Thaldar, 2023). Ubuntu thereby bridges the bottom-up and the top-down structure of other models. Given the strong interest on the African continent in sustainable bioinnovation (Bizoza, 2024), these authentic ethics approaches may hold great promise and the same may be true for value-based approaches in other areas in the world.

Sustainable bioinnovation does not need Social License, it rather needs innovative models for community agreement and responsible decision-making.

## Footnotes

1 The 4A Framework was developed by Lunshof in 1989, in the Netherlands, in collaboration with organizations of patients affected by rare genetic disorders
